# Therapy-related T-lymphoblastic lymphoma following acute myeloid leukemia remission with *IDH2* mutation persistence

**DOI:** 10.1016/j.lrr.2026.100618

**Published:** 2026-10-16

**Authors:** Jonathan J. Cho, Huan-You Wang, Grace Y. Lin, James K. Mangan

**Affiliations:** a Division of Hematology and Cellular Therapy, Department of Medicine, University of California San Diego, La Jolla, CA, 92093, United States; b Department of Internal Medicine, Naval Medical Center San Diego, Uniformed Services University of the Health Sciences, San Diego, CA, 92134, United States; c Department of Pathology, University of California San Diego, La Jolla, CA, 92093, United States

**Keywords:** Secondary T-lymphoblastic lymphoma, IDH2 mutation, Acute myeloid leukemia, Thymoma, Epigenetic modifiers

## Abstract

T-lymphoblastic lymphoma (T-LBL) is an uncommon hematological malignancy that rarely presents as a secondary cancer following chemotherapy. While *IDH2* mutations are commonly identified in acute myeloid leukemia (AML), they were very rarely found in T-LBL. We present a case of an adult male diagnosed with therapy-related T-LBL with an *IDH2* mutation after 3 years of complete remission following chemotherapy for AML with the same *IDH2* mutation, which suggests a dedifferentiated multipotent cancer stem and progenitor cell that may give rise to both the myeloid and lymphoid malignancies.

## 1 Introduction

Secondary cancers have been well-documented as a sequela of chemotherapy and/or radiation therapy with therapy-related acute myeloid leukemia (t-AML) as the most common secondary hematologic malignancy. Therapy-related T- lymphoblastic lymphoma (t-T-LBL) represents <10% of all therapy-related acute lymphoblastic leukemia (t-ALL) cases and is a relatively rare entity [1]. The role of *IDH1* and *IDH2* mutations in AML pathogenesis is well described, but a role for these mutations in T-lymphoblastic leukemia/lymphoma (T-LBL) pathogenesis has only emerged recently [2].

We present a unique case of t-T-LBL following AML complete remission (CR) but with persistent *IDH2* R140Q mutation, which was present in the preceding AML.

## 2 Case report

A 53-year-old male with past medical history of tobacco use and AML in CR for >3 years after treatment with the standard seven days of cytarabine with daunorubicin on days 1 to 3 for induction, followed by high-dose cytarabine consolidation for three cycles, the first two with gemtuzumab ozogamicin, presented to the outside hospital with dyspnea on exertion, and orthopnea. His antecedent AML was characterized as European LeukemiaNet (ELN) favorable risk with mutated *IDH2* (R140Q), mutated *NPM1* but wildtype *FLT3* [3]. His original AML immunophenotype, karyotype/fluorescence *in situ* hybridization (FISH), and gene mutation analyses are summarized in Table 1. On the day of his presentation to the outside hospital, computed tomography (CT) imaging of chest showed bilateral pleural effusion, a large 9 × 4 cm mediastinal mass with associated mediastinal lymphadenopathy, and severe narrowing of superior vena cava as well as right brachiocephalic vein. Transthoracic echocardiography showed moderate pericardial effusion. He received a sternotomy with pericardial window and mediastinal mass debulking at the outside hospital after multiple unsuccessful CT-guided biopsy attempts. Cytomorphologic evaluation of the resected mediastinal mass (Fig. 1) showed a diffuse infiltrate of atypical medium-sized lymphoblasts with moderately condensed nuclear chromatin, small nucleoli and oval to round nuclear contours with starry sky appearance due to increased tingible-body macrophages. Extensive immunohistochemistry showed the atypical lymphoblasts are positive for CD1a, CD2, CD3 (dim), CD4, CD5, CD7, CD8, and TdT with high Ki-67 of >90%, but negative for CD34, B-cell (CD20 and PAX-5) and epithelial (CAM5.2, P40, P63 and Pancytokeratin) markers. The lack of B-cell markers and presence of immature and T-cell markers eliminated the possibility of a B-cell malignancy, including aggressive B-cell malignancies such as Burkitt’s lymphoma, and established the diagnosis of a T-cell malignancy. Markers are most consistent with T-LBL and not thymoma.

**Table 1:** Summaries of immunophenotype, karyotype/FISH, and molecular findings of initial AML and secondary T-lymphoblastic lymphoma with their corresponding specimen type.

Specimen type	Date	Diagnosis	Immunophenotype	Karyotype /FISH	Mutations
Bone marrow	10/2019	AML	86% medium-sized blasts. Positive for CD33, CD45(dim), and CD117; negative for CD34.	46,XY [4] FISH: negative for 5q-,-7/7q-,+8, -13 /13q-,20q-, RUNX1T1::RUNX1, rearrangements of KMT2A, CBFB, and RARA.	Clinically significant from BM and PB: IDH2, NPM1. VUS from BM and PB: DNMT3A. VUS from PB: SMC1A, NOTCH1.
Mediastinal mass	1/2023	T-LBL	100% immature T-cells. Positive for CD1a, CD2, CD3, CD4, CD5, CD7, CD8, TdT; negative for CD20, CD34, PAX5, PAX8. Near 100% Ki-67.	ND	Clinically significant: IDH2, ATM, FBXW7, BCOR.
Pleural fluid	3/2023	T-LBL	98% small to medium sized blasts. Positive for CD1a, CD2(brighter), CD3, CD4, CD5(dim), CD7(brighter), CD8, CD45(dim); negative for CD34. Near 100% Ki-67	ND	ND
Bone marrow	7/2023	T-LBL	No significant increase in T-lymphoblasts or myeloid blasts. 0.7% myeloid blasts with dim partial CD64 expression.	46,XY,+1,der(1;15)(q10;q10)[4]	Clinically significant: IDH2, ATM. VUS: DNMT3A, NOTCH1.

Abbreviation: FISH: fluorescence *in situ* hybridization; AML: acute myeloid leukemia; KMT2A: lysine methyltransferase 2A; CBFB: core-binding factor B; RARA: retinoid receptor alpha; BM: bone marrow; PB: peripheral blood; VUS: variant of undetermined significance; LBL: lymphoblastic lymphoma; ND: not done.

**Fig. 1:**
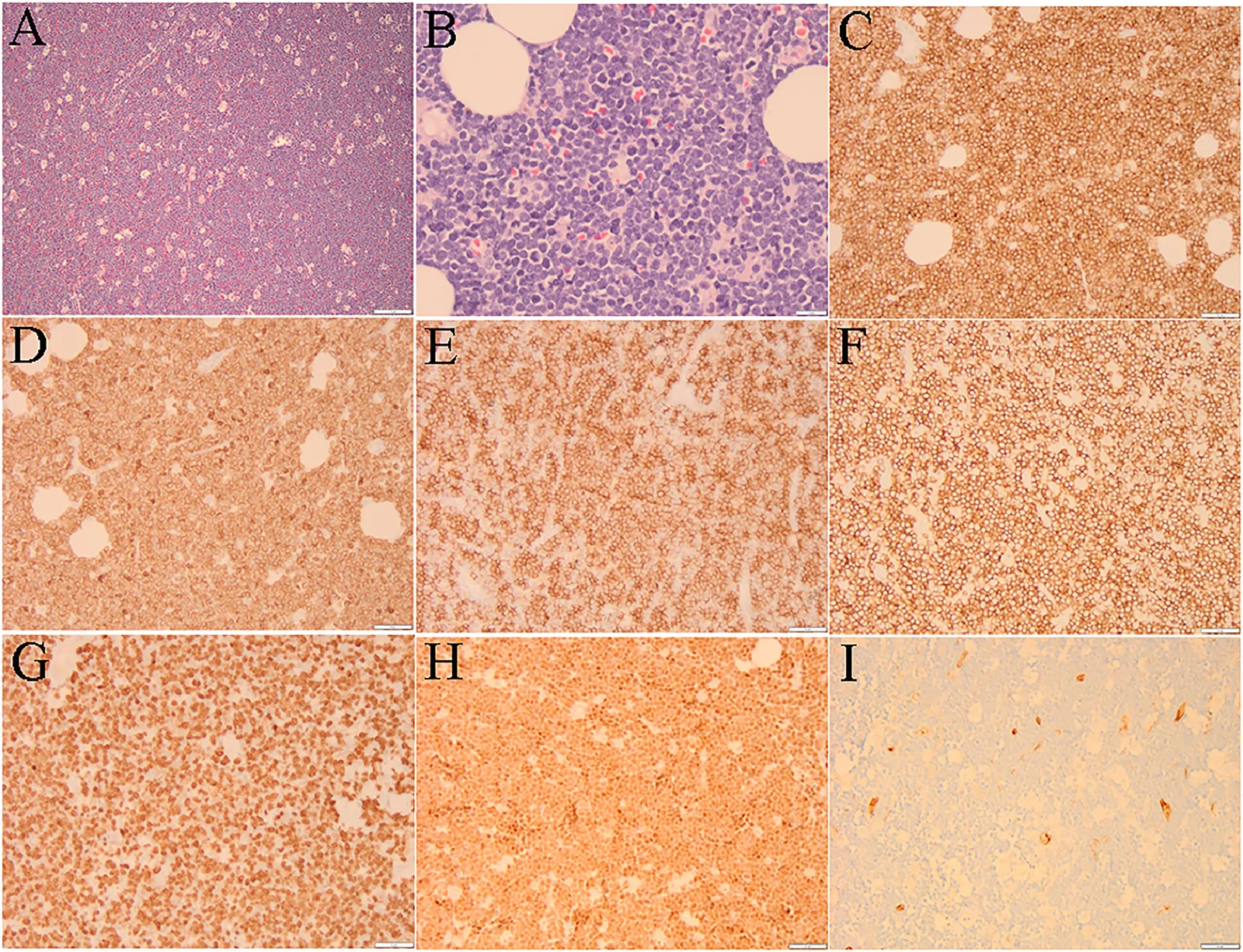
Composite microphotographs of T-lymphoblastic lymphoma from mediastinal mass. There is diffuse infiltrate of lymphoid blasts with starring sky appearance due to presence of tingible-body macrophages (A, original magnification 100x). On high magnification (B, 400x), the lymphoid blasts are small to medium size with mildly condensed nuclear chromatin; these blasts are positive for CD1a (C), CD3 (dim, D), CD4 (E), CD8 (F), high Ki-67 (G), TdT (H), but negative for CD34 (I) (the magnifications for C-I are 200x, 200x, 200x, 200x, 200x, 200x, and 200x, respectively).

Due to concern for superior vena cava syndrome and for further management, he was transferred to our hospital’s intensive care unit and hematologic malignancies service. He was found to have mild anemia (hemoglobin of 11.8 g/dL) but with normal white blood cell (WBC count of 8400/μL), differential count (51% neutrophil, 18% lymphocyte), and platelet of 236,000/μL. A bone marrow (BM) biopsy had been performed prior to transfer to our institution and flow cytometric analysis of BM aspirate showed no significant diagnostic immunophenotypic abnormalities, in particular, no T-lymphoblasts or aberrant myeloid blasts. BM core biopsy showed normal trilineage hematopoiesis with no T-lymphoblasts or increased myeloid blasts. Next generation sequencing was not performed on the bone marrow biopsy. Bone marrow cytogenetics showed the presence of a der (1;15), despite the normal morphology. Flow cytometric analysis of pleural fluid (data not shown) showed ∼98% double CD4+/CD8+ atypical T-lymphoblasts that are negative for CD34 (Table 1). Immunohistochemistry on the cellblock (data not shown) showed the atypical lymphoblasts are positive for CD1a, CD3, CD4, CD8, and TdT with a Ki-67 proliferation index of ∼100%, but negative for B-cell (CD20), granulocytic (CD117 and MPO), immature (CD34) and epithelial (pancytokeratin) markers (Table 1). Similar to the mediastinal biopsy, the pleural fluid showed an identical monoclonal rearrangement of the TCRγ gene. The combined immunohistochemical and molecular profiles are consistent with involvement by T-LBL. Flow cytometric and cytomorphologic evaluation of cerebrospinal fluid showed no myeloid or T-lymphoblasts.

Further molecular studies using institutional-adopted Qiagen 123-gene panel next generation sequencing (NGS) of DNA extracted from the surgically resected mediastinal mass (Table 1) showed mutations in *IDH2* (R140Q), ATM (Q117I*), FBXW7(Q306*), FBWX7 (R465H), and BCOR (E356Dfs*) with variable allele frequencies of 45%, 43%, 27%, 47%, and 88%, respectively, and no mutation was detected in *MLL/KMT2A*. The technology utilized can detect point mutations as well as gene fusions and gene rearrangements.

The patient was emergently consented for hyperfractionated cyclophosphamide, vincristine, doxorubicin, and dexamethasone (hyper-CVAD) chemotherapy. His clinical status markedly improved within 12 h after chemotherapy initiation and he subsequently completed hyper-CVAD through cycle 2A. Bone marrow biopsy performed after hyper-CVAD for his T-LBL are detailed in Table 1 with NGS showing mutations in *IDH2* (R140Q), *ATM* (H2038Y), *DNMT3A* (D529_S535 dup), *NOTCH1* (R2095C) with variable allele frequencies of 37%, 9%, 26%, 48%, respectively. This biopsy showed morphologic remission but persistent derivative chromosome (1;15). Positron emission tomography (PET)–CT showed complete resolution of mediastinal mass except one lesion with fluorodeoxyglucose F18 (FDG) uptake in the oropharynx that was diagnosed as a papilloma on biopsy. He proceeded to reduced-intensity conditioning allogeneic stem cell transplantation (ASCT) from a 10/10 matched-unrelated donor with fludarabine and melphalan conditioning and post-transplant cyclophosphamide, tacrolimus and mycophenolate mofetil for graft-versus-host disease prophylaxis. One-year post-ASCT BM biopsy showed persistent CR with normal, sex-mismatched donor karyotype, >95% and 100% donor DNA in myeloid and T-cell compartments, respectively. PET CT was negative for lymphoma recurrence. Next generation sequencing was not performed on the one-year biopsy but NGS performed on a day +100 biopsy showed no mutations. He remains in CR nearly 3 years after ASCT.

## 3 Discussion

AML pathogenesis involves complex biological mechanisms including deregulated proliferative signaling, block to terminal differentiation, epigenetic modification, and apoptosis resistance. In our patient’s antecedent AML, clinically significant mutations in *IDH2* and *NPM1* were found, both common genomic alterations in AML.

The *IDH* genes encode isocitrate dehydrogenase enzymes that catalyze the oxidative decarboxylation of isocitrate to alpha-ketoglutarate (aKG), and mutations within the IDH genes are commonly found in myeloid malignancies such as AML, myelodysplastic syndrome (MDS) and myeloproliferative neoplasms (MPNs) [5]. The cancer-associated *IDH1* R132 and *IDH2* R140 or R172 mutations in the conserved arginine residues within the enzymatic active site cause a reverse reaction where aKG is reduced to 2-hydroxyglutarate (2HG). The oncometabolite 2HG competitively inhibits aKG-dependent enzymes such as the TET family and lysine demethylases, leading to a hypermethylated phenotype in AML, which means that the IDH1/2 mutations are epigenetic modifiers [4]. Although *IDH1* or *IDH2* mutations are found in 8% or 12% of AML, respectively [5], they are present in only 2% and 3%, respectively, among 1085 T-ALLs analyzed [6]; however, IDH2 mutations increase to 7% in T-ALLs among elderly and therapy-related patients [2], such as the case with our patient. The finding of the same *IDH2* (R140Q) mutation in our patient’s T-LBL strongly suggests he may harbor a highly dedifferentiated cancer stem cell that gave rise to AML and subsequent t-T-LBL.

In our patient’s antecedent AML, mutations in IDH2, NPM1, DMNT3A, and SMC1A genes were found, which are consistent with common genomic alterations in AML. Mutations associated with clonal hematopoiesis of indeterminate potential (CHIP) are hypothesized to be early events in the development of AML and may be associated with mutations in epigenetic modifier genes such as DNMT3A (seen in our patient), TET2, and ASXL1 [7,8]. The same DNMT3A mutation was found in our patient’s antecedent AML and bone marrow aspirate after treatment for t-T-LBL, in the setting of a morphologically normal bone marrow. Thus, a mutation associated with clonal hematopoiesis was present in his original marrow diagnostic of AML and a subsequent bone marrow biopsy that was morphologically normal but performed after presentation with and treatment for t-T-LBL. The role that clonal hematopoiesis plays in the pathogenesis of t-T-LBL remains unclear. Even more fascinating was the fact that the IDH2 mutation present in the original AML bone marrow biopsy, and the subsequent-sT-LBL mass, was also present in the morphologically normal bone marrow biopsy performed after treatment for t-T-LBL. The IDH2 mutation is thus present in a myeloid clone, a lymphoid clone, and in a morphologically normal bone marrow, representing clonal hematopoiesis that apparently has potential to differentiate along both myeloid and lymphoid pathways. Notably, the NPM1 mutation was only present in the patient’s AML and was not seen in either sequencing of the T-LBL or in the biopsies after treatment of the T-LBL.

Increased risk and incidence of secondary cancers after chemotherapy and/or radiotherapy are well documented, but therapy-related T-LBL is rare. In a series of 116 therapy related ALL patients, only 8% had a T-cell immunophenotype, but the patients in this series were enriched in myeloid mutations similar to our patient [1]. Abnormalities of chromosome 5 and/or 7 are associated with t-ALL [1,9]; however, cytogenetic analysis was not performed on the t-T-LBL of our patient to compare this case with previous studies. Treatment with topoisomerase II inhibitors for antecedent malignancy is associated with t-AML and t-ALL [1,3,9], and, in our patient’s case, suggest association with t-T-LBL. Our patient’s development of t-T-LBL in <4 years after receiving daunorubicin as part of his antecedent AML treatment regimen is consistent with shorter latency of patients who received topoisomerase II inhibitors (median, 4.7 years; range, 0.5–16) compared to those who did not receive these agents (median, 6.3 years; range, 1–35) in one retrospective study [1]. T-ALL is associated with unfavorable prognosis and inferior survival, but the association between cytotoxic therapy exposure or hematopoietic cell transplantation and survival differs amongst previous studies [1,9].

The current case highlights a rare case of sT-LBL following AML CR but with persistent identical *IDH2* mutation, which suggests a key role of the *IDH2* mutation in hematologic malignancy predisposition and risk, regardless of myeloid or T-lymphoid lineage.

## Disclaimer

The views expressed in this article are those of the authors and do not necessarily reflect the official policy or position of the Department of the Navy, DoD, nor the U.S. Government. The views expressed are solely those of the authors and do not reflect the official policy or position of the U.S. Army, U.S. Navy, U.S. Air Force, the DoD, or the U.S. Government.

## Ethics declaration

Written informed consent to take part in the study and to publish the article has been obtained from all participants or their legal representatives. The privacy rights of participants have been observed.

This study was performed in compliance with relevant laws, regulatory frameworks and guidelines where the research took place. Ethics committee approval was not required under relevant laws and institutional guidelines. Informed consent was obtained for the publication of this case report. This case report is exempt from the institutional review board.

This research follows the CARE guidelines and the CARE checklist.

## Funding sources

JJC: none.

HYW: none.

GYL: none.

JKM: none.

## CRediT authorship contribution statement

**Jonathan J. Cho:** Writing – review & editing, Writing – original draft, Visualization, Validation, Supervision, Software, Resources, Project administration, Methodology, Investigation, Funding acquisition, Formal analysis, Data curation, Conceptualization. **Huan-You Wang:** Writing – review & editing, Writing – original draft, Visualization, Validation, Supervision, Software, Resources, Project administration, Methodology, Investigation, Funding acquisition, Formal analysis, Data curation, Conceptualization. **Grace Y. Lin:** Writing – review & editing, Writing – original draft, Visualization, Validation, Supervision, Software, Resources, Project administration, Methodology, Investigation, Funding acquisition, Formal analysis, Data curation, Conceptualization. **James K. Mangan:** Writing – review & editing, Writing – original draft, Visualization, Validation, Supervision, Software, Resources, Project administration, Methodology, Investigation, Funding acquisition, Formal analysis, Data curation, Conceptualization.

## Declaration of competing interest

The authors declare that they have no known competing financial interests or personal relationships that could have appeared to influence the work reported in this paper.
